# Perinatal *Ureaplasma* Exposure Is Associated With Increased Risk of Late Onset Sepsis and Imbalanced Inflammation in Preterm Infants and May Add to Lung Injury

**DOI:** 10.3389/fcimb.2019.00068

**Published:** 2019-04-02

**Authors:** Kirsten Glaser, Anna Gradzka-Luczewska, Marta Szymankiewicz-Breborowicz, Natalia Kawczynska-Leda, Birgit Henrich, Ana Maria Waaga-Gasser, Christian P. Speer

**Affiliations:** ^1^University Children's Hospital, University of Wuerzburg, Wuerzburg, Germany; ^2^Department of Neonatology, Poznan University of Medical Sciences, Poznan, Poland; ^3^Institute of Medical Microbiology and Hospital Hygiene, University Clinic of Heinrich-Heine University Duesseldorf, Duesseldorf, Germany; ^4^Department of Surgery I, Molecular Oncology and Immunology, University of Wuerzburg, Wuerzburg, Germany

**Keywords:** *Ureaplasma parvum*, *Ureaplasma urealyticum*, preterm infants, VLBW, bronchopulmonary dysplasia, late onset sepsis, neonatal outcome, inflammation

## Abstract

**Background:** Controversy remains concerning the impact of *Ureaplasma* on preterm neonatal morbidity.

**Methods:** Prospective single-center study in very low birth weight infants <30 weeks' gestation. Cord blood and initial nasopharyngeal swabs were screened for *Ureaplasma parvum* and *U. urealyticum* using culture technique and polymerase chain reaction. Neonatal outcomes were followed until death or discharge. Multi-analyte immunoassay provided cord blood levels of inflammatory markers. Using multivariate regression analyses, perinatal *Ureaplasma* exposure was evaluated as risk factor for the development of bronchopulmonary dysplasia (BPD), other neonatal morbidities until discharge and systemic inflammation at admission.

**Results:** 40/103 (39%) infants were positive for *Ureaplasma* in one or both specimens, with *U. parvum* being the predominant species. While exposure to *Ureaplasma* alone was not associated with BPD, we found an increased risk of BPD in *Ureaplasma*-positive infants ventilated ≥5 days (OR 1.64; 95% CI 0.12–22.98; *p* = 0.009). Presence of *Ureaplasma* was associated with a 7-fold risk of late onset sepsis (LOS) (95% CI 1.80–27.39; *p* = 0.014). Moreover, *Ureaplasma*-positive infants had higher I/T ratios (*b* 0.39; 95% CI 0.08–0.71; *p* = 0.014), increased levels of interleukin (IL)-17 (*b* 0.16; 95% CI 0.02–0.30; *p* = 0.025) and matrix metalloproteinase 8 (*b* 0.77; 95% CI 0.10–1.44; *p* = 0.020), decreased levels of IL-10 (*b* −0.77; 95% CI −1.58 to −0.01; *p* = 0.043) and increased ratios of Tumor necrosis factor-α, IL-8, and IL-17 to anti-inflammatory IL-10 (*p* = *0.0*03, *p* = 0.012, *p* < 0.001).

**Conclusions:** Positive *Ureaplasma* screening was not associated with BPD. However, exposure contributed to BPD in infants ventilated ≥5 days and conferred an increased risk of LOS and imbalanced inflammatory cytokine responses.

## Introduction

Although substantial therapeutic advances have continously improved the survival of preterm infants, the incidence of neonatal morbidity and sequelae has not declined (Stoll et al., 2015). This is particularly due to the heightened susceptibility of very immature preterm infants to severe infections and major morbidities, such as bronchopulmonary dysplasia (BPD) (Stoll et al., 2015). Colonization with *Ureaplasma* species (spp.) has been associated with increased risk of BPD in preterm infants (Viscardi and Hasday, 2009; Lowe et al., 2014), and there is some evidence of additional implication in the pathogenesis of intraventricular hemorrhage (IVH), necrotizing enterocolitis (NEC) and retinopathy of prematurity (ROP) (Viscardi et al., 2008; Kasper et al., 2011; Silwedel et al., 2017). *Ureaplasma* spp. have been frequently isolated from amniotic fluid, cord blood and respiratory tract samples of preterm infants who later developed BPD (Viscardi and Hasday, 2009). *Ureaplasma* respiratory tract colonization appears to contribute to pulmonary inflammation and altered lung development (Viscardi and Hasday, 2009). Intraamniotic detection of *Ureaplasma* spp. was shown to be paralleled by intrauterine and fetal inflammatory response and increased risk of adverse pulmonary and neurologic outcome in preterm infants (Berger et al., 2009; Kasper et al., 2011; Glaser and Speer, 2015; Sweeney et al., 2017). However, the clinical relevance of detecting *Ureaplasma* spp. in microbiological specimens of preterm infants remains subject of discussion. *Ureaplasma parvum* (serovars 1, 3, 6, 14) and *U. urealyticum* (serovars 2, 4, 5, 7–13) are generally regarded as commensal bacteria being isolated from 40 to 80% of urogenital tract samples of women of reproductive age (Waites et al., 2005; Sweeney et al., 2017). For reasons of low pathogenicity in children and adults, the presence of *Ureaplasma*-driven inflammation and its impact on neonatal morbidity have been discussed controversially (Volgmann et al., 2005; Glaser and Speer, 2015). The present study aimed to investigate whether early life exposure to *Ureaplasma* spp. is associated with (i) the development of BPD, (ii) the development of other neonatal morbidities until discharge, and (iii) systemic inflammation at admission in a cohort of very low birth weight infants (VLBW, i.e., birth weight < 1,500 g) born at <30 weeks' gestational age (GA).

## Methods

### Study Outline

**Figure 1:**
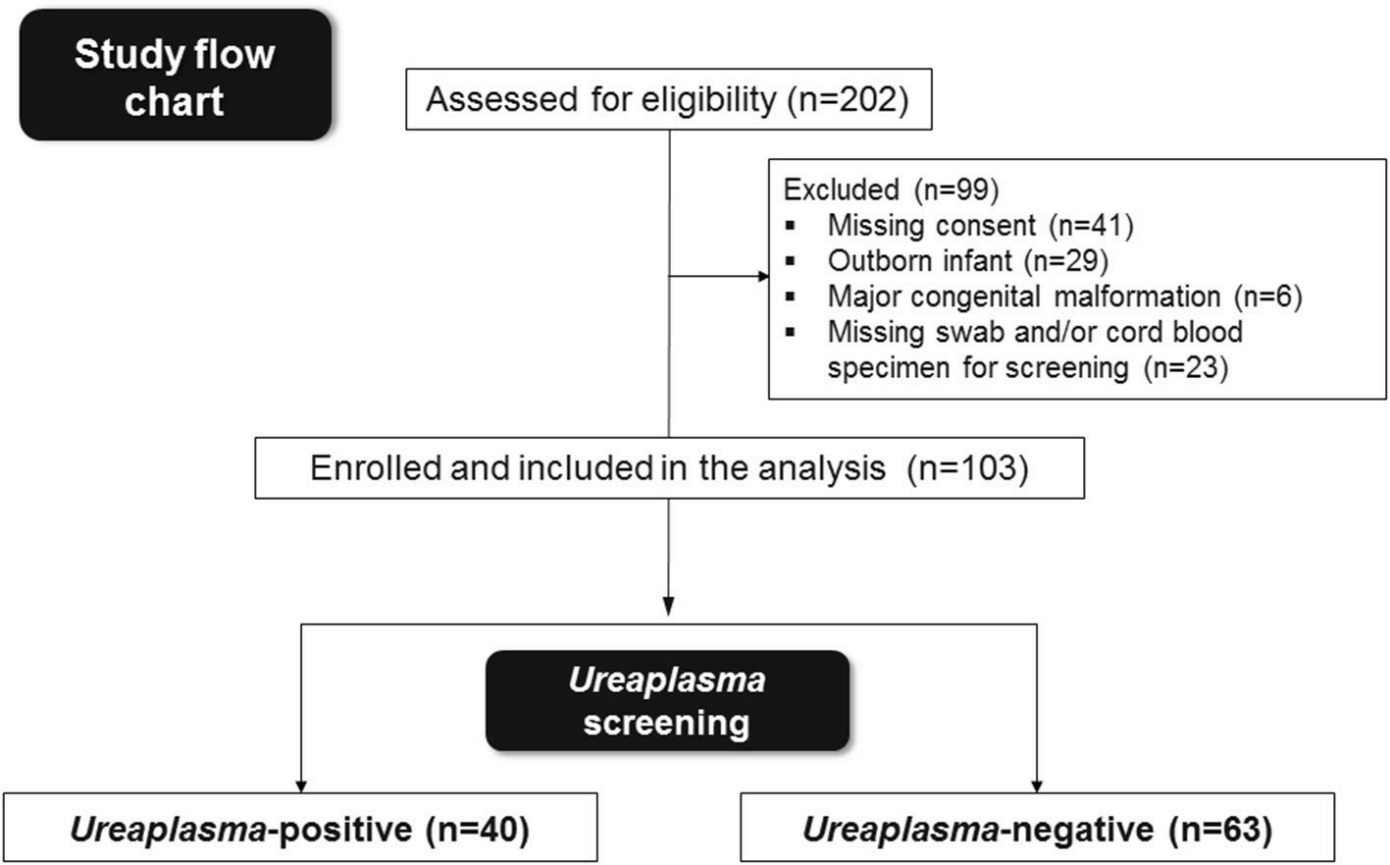
Summary of preterm infants eligible, enrolled in the study and included in the final analysis.

Preterm infants with GA <30 weeks and birth weight <1,500 g admitted to the tertiary neonatal intensive care unit at the Children's Hospital of Poznan, University of Medical Sciences, Poland, were eligible for this prospective study conducted between May 2014 and December 2015. Outborn infants as well as infants with major congenital malformations, lack of informed consent or absence of specimens for *Ureaplasma* screening and/or analysis of cord blood inflammatory markers were excluded. Among 202 infants eligible, 103 met the primary inclusion criteria (Figure 1). Infants were categorized as *Ureaplasma*-positive and negative depending on initial screening. Infant data were entered into a computerized data sheet, verified by two different investigators. Neonatal outcomes were followed until death or discharge.

### Outcome Definitions

BPD, the primary endpoint, was defined as requirement of oxygen therapy for at least 28 days and classified according to the need of supplemental oxygen at 36 weeks' postmenstrual age (Jobe and Bancalari, 2001). Predefined secondary endpoints comprised respiratory distress syndrome (RDS) ≥ grade 3, IVH ≥ grade 3, periventricular leukomalacia (PVL), NEC ≥ stage 2, ROP ≥ stage 3, late onset sepsis (LOS), meningitis and death prior to discharge. Diagnosis of RDS referred to clinical and radiographic features, and the requirement of supplemental oxygen. IVH was diagnosed by means of cranial ultrasound and graded according to the Papile classification (Papile et al., 1978). PVL was defined as bilateral cystic lesions in periventricular areas documented in coronal and parasagittal ultrasound scans performed > 28 days after birth (Perlman et al., 1996). NEC was diagnosed by clinical and radiological signs according to Bell's staging (Bell, 1978). ROP was determined following the international classification (International Committee for the Classification of Retinopathy of Prematurity, 2005). Diagnosis of LOS required clinical and laboratory signs of systemic infection at >72 h of age, positive blood culture and antibiotic treatment ≥ 5 days. Meningitis was defined by clinical and laboratory signs of systemic infection, abnormal cerebrospinal fluid parameters, and positive cerebrospinal fluid culture.

### Sample Collection and Laboratory Methods

Umbilical cord blood samples were taken immediately after delivery using an aseptic technique. The needle puncture site was disinfected with a 0.1% aqueous solution of octenidine with phenoxyethanol (Octenisept®, Schuelke & Mayr GmbH, Germany) applied by means of a spray and saturated swabs thereafter. The site was allowed to dry for 1 min before cord blood was drawn using a sterile, unheparinized syringe. For *Ureaplasma* culture, 0.5 mL cord blood was immediately inoculated into 2 mL 10B broth (Thermo Fisher Scientific, Germany). Nasopharyngeal swabs obtained within 2 h after birth using sterile transport swabs (Copan, Italy) were also immediately placed in the commercially available ready-to-use tubes providing 2 mL of 10B broth. Swab and liquid cultures were incubated at 37°C in 5% CO_2_/air for 10 days and monitored daily. If color change occurred, inoculums of 0.2 mL were plated on A8 agar to confirm positive broth. All cord blood samples and swabs were additionally investigated for DNA of *U. parvum* and *U. urealyticum* using commercially available kits for DNA extraction and amplification according to the manufacturer's protocol (AmpliSens® DNA-sorb-B, AmpliSens® *U. parvum/U. urealyticum*-FRT; Ecoli Ltd., Slovak Republic). An *mba*-based real-time PCR approach was used to discriminate *U. parvum* serovars (Otgonjargal et al., 2018). Routine cord blood and nasopharyngeal swab cultures were assessed using the Bactec Peds Plus/F blood culture system (bioMérieux, Poland), selective agars and the Vitek® MS system (bioMérieux).

Cord blood leukocyte counts, immature/total neutrophil (I/T) ratios and C-reactive protein levels were analyzed within 2 h after birth. For quantification of TNF-α, IL-1β, IL-8, IL-12, IL-17, IL-10, IL-1ra, IFN-γ, IP-10, MMP-8/9, MIP-1α/β, MCP-1, VEGF, G-CSF, ICAM-1, and VCAM-1, serum aliquots of ≥0.2 mL were stored at −80°C until analysis. Multianalyte immunoassay was carried out using Luminex® multiplex kits and the xPonent® software (Merck group, Germany) as described before (Glaser et al., 2017, 2018a,b).

### Maternal Screening and Placental Pathology

Maternal vaginal screening was assessed at admission as part of routine clinical care. Bacterial vaginosis was defined according to the Nugent criteria (Nugent et al., 1991). Placental examination was part of the research study and was conducted after written informed consent was obtained. Histopathological investigation was performed by 2 independent pathologists at the Institute of Pathology, Poznan University of Medical Sciences, following a published protocol (Rogers et al., 2002).

### Statistical Analyses

Sample size was determined using an approximate sample size formula for studies with dichotomous outcomes (Schulz and Grimes, 2005). Based on preliminary data reporting on incidences of BPD in *Ureaplasma* colonized preterm infants (Lowe et al., 2014) and baseline BPD rates of about 25% in infants <32 weeks' GA in Poland (Fanaroff et al., 2007; Gortner et al., 2011), we calculated that minimum 38 infants per study group needed to be enrolled and included in the final analysis to detect a 2-fold risk of BPD with α = 0.05 and power.80. Infant characteristics were analyzed descriptively, and tested for differences using *X*^2^, Fisher's exact test and Cramér's *V* for qualitative variables, Q-Q plots, unpaired *t*, and Mann-Whitney *U* test for quantitative variables. Bivariate analyses using *X*^2^, Fisher's exact test and Cramér's *V* and multivariate logistic regressions were performed to test for associations between *Ureaplasma* exposure and primary and secondary outcomes. Covariates offered to the models were GA, birth weight, male sex, singleton status, antenatal corticosteroids, antenatal antibiotics, antenatal macrolides, preterm premature rupture of membranes >12 h, histologic chorioamnionitis, vaginal delivery, vaginal *Ureaplasma*/GBS/other bacteria/*Candida*, surfactant treatment, ventilation days, supplemental oxygen days, patent ductus arteriosus requiring treatment as well as presence of and duration of central venous lines. Covariates were included if significant at *p* < 0.10 in preceding analyses using *X*^2^, Fisher's exact test and Cramér's *V*, Q-Q plots, unpaired *t* and Mann-Whitney *U*. Subgroup analyses were performed on *Ureaplasma*-positive infants and Ureaplasma-negative infants, respectively, ventilated < or ≥5 days using the same approach. Corresponding bivariate and stepwise multivariate linear regression analyses examined the relationship between *Ureaplasma* exposure and cord blood inflammatory markers. A *p-*value < 0.05 was considered significant. Analyses were performed using the Statistical Package of Social Sciences (SPSS) software, version 24 (IBM, Germany).

## Results

**Figure 2:**
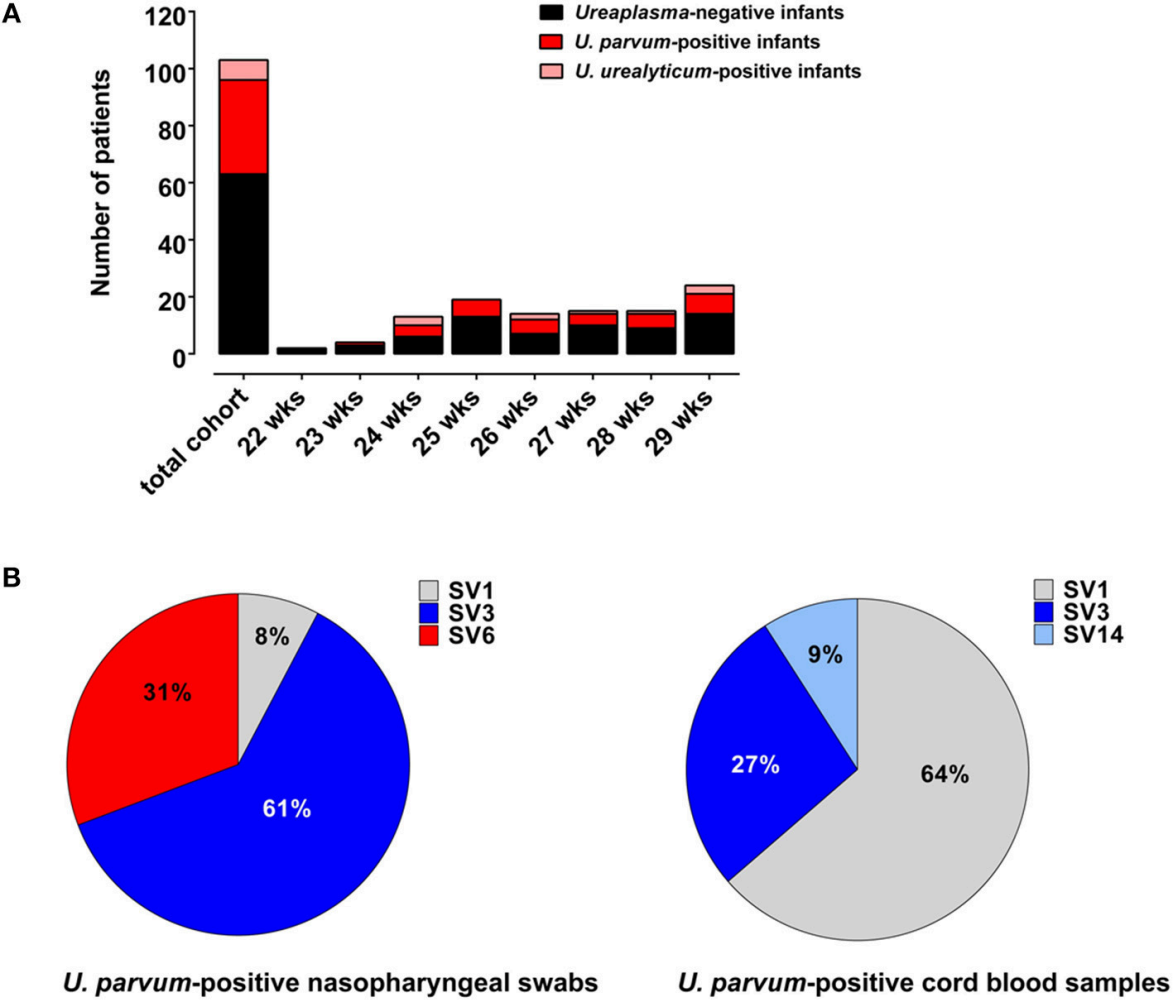
**(A)** Numbers of *Ureaplasma*-negative infants as well as *U. parvum*-positive and *U. urealyticum*-positive infants given for each week of gestation (assessed as completed weeks). **(B)** Distribution of *U. parvum* serovars in *Ureaplasma*-positive swab and cord blood specimens.

During the study period, 202 VLBW infants born at <30 weeks GA were admitted to the Poznan NICU. One hundred and three infants met the inclusion criteria and were included in the final analysis (Figure 1). Among these, 40 (39%) were culture/PCR positive for *Ureaplasma* spp. in one or both specimens and 63 (61%) were *Ureaplasma-*negative. The *Ureaplasma-*positive group comprised 32 infants with positive nasopharyngeal swabs, 16 infants with cord blood positive for *Ureaplasma* and 8 infants with detection of *Ureaplasma* spp. in both specimens. Quantitative real-time PCR detected *Ureaplasma* spp. in 40/40 *Ureaplasma*-positive study infants, culture technique spotted *Ureaplasma* in 11/40. Being detected in 33/40 (83%) *Ureaplasma*-positive infants, *U. parvum* was the predominant species. *U. urealyticum* was found in 7/40 infants, 3/40 infants carried both species. Differentiation of *U. parvum* serovars revealed different distribution patterns in swab and cord blood specimens (Figure 2). Serovar differentiation of *U. urealyticum* was not available. Detection of *Ureaplasma* was more frequent in infants with a history of vaginal delivery (*p* = 0.024) and maternal vaginal colonization with *Ureaplasma* (*p* = 0.003) and *Candida* spp. (*p* = 0.026) (Table 1). Additionally, *Ureaplasma*-positive and negative study infants differed in terms of central venous lines present, with a higher number of infants with a central venous catheter in the *Ureaplasma*-negative group (*p* = 0.006). Clinical characteristics otherwise did not differ (Table 1). None of the infants had a positive routine cord blood culture. Routine cultures of initial nasopharyngeal swabs detected *Candida* spp., *Staphylococcus epidermidis, Staphylococcus haemolyticus, Staphylococcu*s *hominis, Streptococcus anginosus* and *Gardnerella vaginalis* in 23 and 22% of infants, respectively (Table 1).

**Table 1:** Prenatal, perinatal, and postnatal characteristics of study infants.

**Characteristic**	***Ureaplasma***		***p* value^a^**
	**Positive infants (*n* = 40)**	**Negative infants (*n* = 63)**	
Gestational age (weeks), mean (range)	26 (23–29)	26 (22–29)	0.919
Birth weight (g), mean (range)	912 (460–1,490)	872 (440–1485)	0.394
Sex (male), *n* (%)	23 (58%)	42 (67%)	0.488
Singleton, *n* (%)	31 (78%)	48 (76%)	>0.999
Antenatal steroids, *n* (%)	38 (95%)	61 (97%)	>0.999
Antenatal antibiotics, *n* (%)	27 (68%)	45 (71%)	0.889
Antenatal macrolides, *n* (%)	21 (53%)	30 (48%)	0.416
PPROM > 12 h, *n* (%)	13 (33%)	22 (35%)	>0.999
Histologic chorioamnionitis, *n* (%)^b^	21 (53%)	28 (44%)	0.405
Vaginal delivery, *n*(%)	20 (50%)	16 (25%)	0.023^*^^†^
Vaginal *Ureaplasma, n* (%) ^c^	21 (53%)	15 (24%)	0.004^**^^‡^
Vaginal GBS, *n* (%)^d^	3 (8%)	3 (5%)	0.502
Bacterial vaginosis, *n* (%)^e^	1 (3%)	2 (3%)	>0.999
Vaginal *Candida, n* (%)	13 (33%)	8 (13%)	0.020^*^^#^
Other microbes nasopharyngeally, *n* (%)	9 (23%)	14 (22%)	>0.999
Surfactant administration, *n* (%)	27 (68%)	44 (70%)	0.731
Ventilation days, mean (range)	19.1 (0–149)	20.2 (0–84)	0.360
Supplemental oxygen days, mean (range)	14.8 (0–35)	16.9 (0–60)	0.677
PDA requiring treatment, *n* (%)	9 (23%)	24 (38%)	0.201
Presence of central venous line, *n* (%)	25 (63%)	55 (87%)	0.012^*^^^##^^
Central venous line days, mean (range)	11.2 (2–21)	12.7 (3–37)	0.401

a *Pearsons's chi-square (X^2^) and Fisher's exact test for qualitative variables. Q-Q plots, unpaired t and Mann-Whitney U test for quantitative variables*.

* p < 0.05,

** *p < 0.01*.

† Cramér‘s V 0.256 (p 0.014), Cramér‘s V 0.417 (p 0.003),

# Cramér‘s V 0.241 (p 0.011),

## *Cramér‘s V 0.322 (p 0.01)*.

b *Histopathological examination performed in 89 placentas*.

c *Data available in 89 mothers*.

d *Data available in 91 mothers*.

e *Nugent score ≥ 7*.

*GBS, group B Streptococcus; PDA, patent ductus arteriosus; PPROM, preterm premature rupture of membranes*.

Bivariate and stepwise multivariate logistic regression analyses were used to test the primary hypothesis of increased BPD rates in *Ureaplasma*-positive infants and the association between *Ureaplasma* exposure and secondary endpoint measures. On bivariate analysis, BPD rates did not differ among both study groups (*p* = 0.218) (Table 2). Analyses including BPD severity (*p* = 0.629) and the compound measure moderate/severe BPD did not detect a significant correlation either (*p* = 0.374). Covariates independently associated with BPD were ventilation days (*p* = 0.039) and supplemental oxygen days (*p* = 0.032). Subgroup analysis was performed comparing *Ureaplasma*-positive infants with a history of < 5 days of mechanical ventilation (*n* = 20) with *Ureaplasma*-positive infants ventilated ≥5 days (*n* = 20), to examine whether perinatal *Ureaplasma* exposure interacts with mechanical ventilation. Bivariate analyses documented increased rates of BPD in *Ureaplasma*-positive infants ventilated ≥5 days (87 vs. 28%, *p* = 0.001), and adjusted analysis confirmed increased odds of BPD in infants with positive *Ureaplasma* screening (OR 1.64; 95% CI 0.12–22.98; *p* = 0.009). Covariates included in the model were GA, birth weight, male sex, vaginal delivery, antenatal antibiotics, vaginal *Ureaplasma*, RDS ≥grade 3, surfactant administration, ventilation and supplemental oxygen days. Birth weight was an independent predictor of BPD in this model (OR 0.99; 95% CI 0.98–1.00; *p* = 0.007). Analyses in the *Ureaplasma*-negative group documented BPD rates of 70% in infants ventilated ≥5 days compared to 48% in infants ventilated < 5 days (*p* = 0.156).

**Table 2:** Bivariate analyses testing for associations between perinatal exposure to *Ureaplasma* and primary and secondary outcome measures.

**Outcome**	***Ureaplasma***		***p* value^a^**
	**Positive infants (*n* = 40)**	**Negative infants (*n* = 63)**	
BPD total^b^	18/36 (50%)	32/56 (57%)	0.218
BPD^b^			0.629
– Mild	6/36 (17%)	12/56 (21%)	
– Moderate	8/36 (22%)	12/56 (21%)	
– Severe	4/36 (11%)	8/56 (14%)	
RDS ≥ grade 3	14 (35%)	28 (44%)	0.481
IVH ≥ grade 3	11 (28%)	17 (27%)	0.949
PVL^c^	5/36 (14%)	10/56 (18%)	0.487
NEC ≥ stage 2	4 (10%)	9 (14%)	0.536
ROP ≥ stage 3^c^	3/36 (8%)	7/56 (13%)	0.478
Late onset sepsis	10 (25%)	6 (10%)	0.060^†^
Meningitis	0 (0%)	2 (3%)	0.288
Death prior to discharge	4 (10%)	8 (13%)	0.602

*Values are expressed as numbers and proportions*.

a *Pearsons's chi-square (X^2^), Fisher's exact test and Cramér's V*.

† *Cramér's V 0.211 (p 0.048)*.

b *Diagnosis assessed in 92 infants at 36 weeks PMA—based upon the criterion of supplemental oxygen for at least 28 days and classified according to the amount of supplemental oxygen at 36 weeks PMA (11 infants died prior to 28 days of life and prior to 36 weeks PMA)*.

c *Data available in 92 infants*.

*BPD, bronchopulmonary dysplasia; IVH, intraventricular hemorrhage; PVL, periventricular leukomalacia; PMA, postmenstrual age; NEC, necrotizing enterocolitis; RDS, respiratory distress syndrome; ROP, retinopathy of prematurity*.

Testing the association between perinatal *Ureaplasma* exposure and secondary outcomes, we found a tendency toward increased rates of LOS in *Ureaplasma-positive* infants (Table 2). On multivariate regression adjusting for confounding variables, detection of *Ureaplasma* spp. was associated with a 7-fold risk of LOS (Table 3). There was no difference in the onset of sepsis among *Ureaplasma*-positive and negative study infants [mean day of life 17.0 (range 6–51) vs. 19.8 (7–52), *p* = 0.355]. Moreover, both groups did not differ in terms of exposure to a prior empiric antibiotic therapy at admission, that was mostly limited to the first 48–72 h of life. No infant had more than one episode of sepsis. Blood cultures of *Ureaplasma*-positive infants with LOS were positive for *Escherichia coli* (4 infants), *Staphylococcus haemolyticus* (3 infants), *Staphylococcus aureus* and *Staphylococcus epidermidis*. Infecting organisms in *Ureaplasma*-negative infants *were Escherichia coli, Enterococcus faecalis, Acinetobacter baumanii, Streptococcus agalactiae, Staphylococcus haemolyticus, and Candida albicans*. *Ureaplasma*-positive infants with and without LOS did not significantly differ in primary or secondary outcome measures or in terms of species and serovar isolated. *U. parvum* was present in 70% of infants with LOS (serovar 3: 57%, serovar 6: 28%, serovar 1: 14%). In one infant both species were detected.

**Table 3:** Stepwise multivariate logistic regression analysis examining the correlation between *Ureaplasma* exposure and late onset sepsis.

**Outcome**	**Late onset sepsis**	***p*-value^a^**
**Independent variables**	**OR (95% CI)**	
Presence of *Ureaplasma* spp.	6.955 (1.801–27.390)	0.014^*^
Sex (male)	0.297 (0.078–1.133)	0.076
Vaginal delivery	0.201 (0.039–1.019)	0.053
Vaginal *Ureaplasma* spp.	0.981 (0.231–4.169)	0.981
Vaginal *Candida* spp.	0.610 (0.110–3.390)	0.572
Presence of central venous line	0.630 (0.146–2.714)	0.535

a *Covariates offered to the model were independent variables significant at p < 0.10 in preceding bivariate analyses*.

* *p < 0.05*.

*CI, confidence interval; OR, odds ratio*.

Exposure to *Ureaplasma* spp. was not associated with the development of RDS ≥grade 3, IVH ≥grade 3, PVL, NEC ≥stage 2, ROP ≥stage 3, meningitis and death prior to discharge (Table 2; characteristics of all infants who died prior to discharge and causes of death given in Supplementary Table S1). However, subgroup analyses comparing *Ureaplasma*-positive infants ventilated <5 days with those ventilated ≥5 days revealed increased rates of IVH ≥grade 3 and PVL in the latter (IVH ≥grade 3: 0 vs. 55%, *p* < 0.001; PVL: 0 vs. 29%, *p* = 0.016). On multivariate regressions controlling for GA, birth weight, vaginal *Ureaplasma*, ventilation days, RDS ≥grade 3 and patent ductus arteriosus requiring treatment and GA, birth weight, surfactant administration and ventilation days, respectively, co-exposure to *Ureaplasma* and mechanical ventilation ≥5 days was associated with increased odds of IVH ≥grade 3 (OR 3.36; 95% CI 0.70–16.617; *p* = 0.030). The correlation with PVL was no longer significant (OR 0.1; 95% CI 0.01–1.09; *p* = 0.059). Low GA (OR 0.27; 95% CI 0.08–0.93; *p* = 0.037) and RDS ≥grade 3 (OR 25.53; 95% CI 1.18–554.41; *p* = 0.040) were independent risk factors for IVH ≥grade 3. Analyses in the *Ureaplasma*-negative group revealed rates of IVH ≥grade 3 of 18% in infants ventilated < 5 days and 32% in infants with a history of mechanical ventilation ≥5 days (*p* = 0.205) as well as similar rates of PVL in both subgroups (13 vs. 15%, *p* = 0.663).

**Figure 3:**
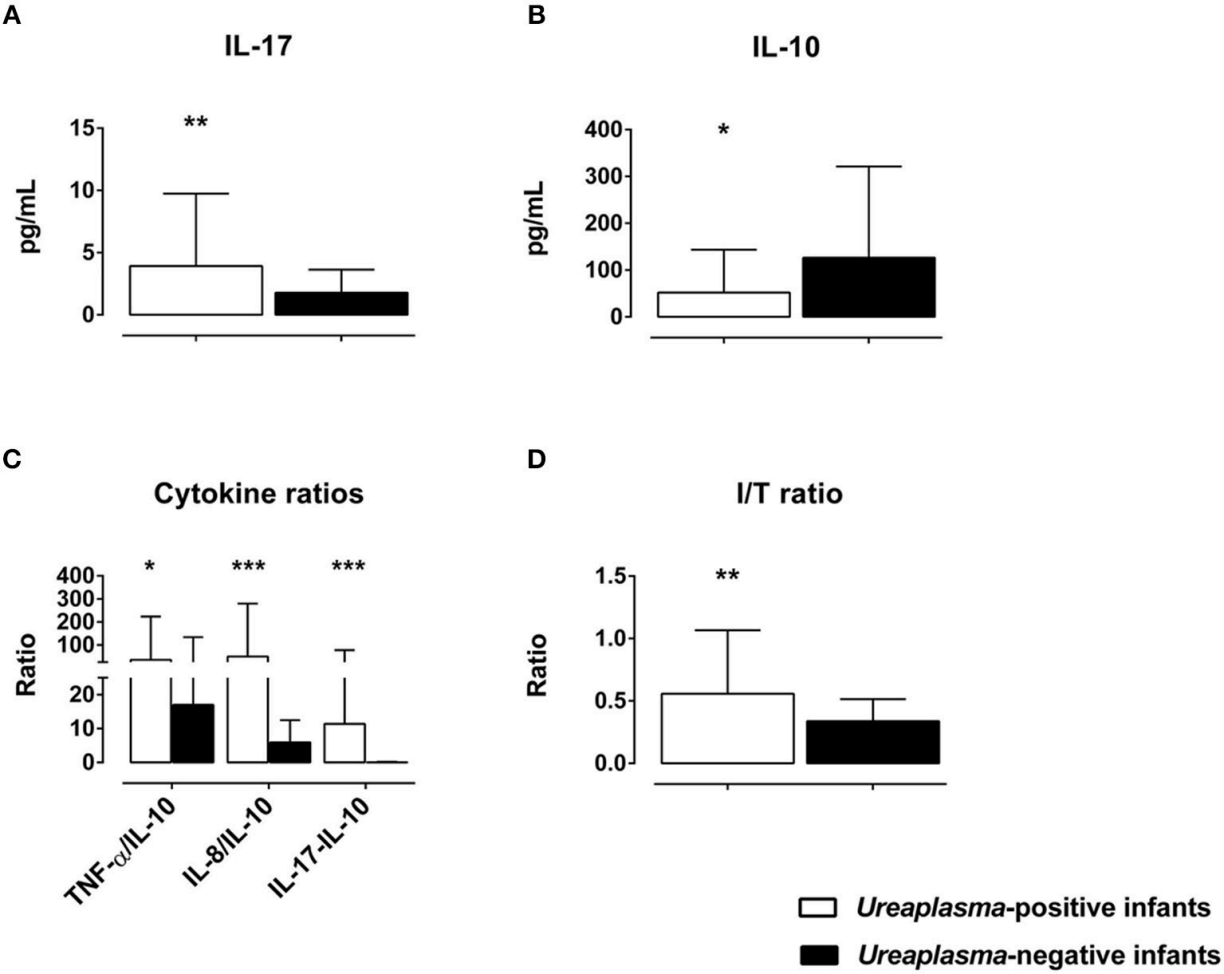
Differences in cord blood serum levels of IL-17 **(A)** and IL-10 **(B)**, ratios of pro-inflammatory cytokines to anti-inflammatory IL-10 **(C)** and I/T ratios **(D)** among *Ureaplasma*-positive and *Ureaplasma*-negative infants on bivariate analysis (*p < 0.05, **p < 0.01, ***p < 0.001).

Cord blood levels of inflammatory markers were compared among *Ureaplasma*-positive and *Ureaplasma*-negative infants. We found higher I/T ratios and increased levels of IL-17, but decreased levels of IL-10 and increased ratios of pro-inflammatory cytokines to anti-inflammatory IL-10 in the former group (Figure 3). Moreover, *Ureaplasma*-positive infants had higher levels of MMP-8 (*p* = 0.024) and ICAM-1 (*p* = 0.041) and showed a trend toward decreased levels of IL12p40 in cord blood (*p* = 0.089). Stepwise multivariate linear regressions adjusting for confounding confirmed an association between positive *Ureaplasma* screening and increased I/T ratios and levels of IL-17 and MMP-8, decreased levels of IL-10 and increased ratios of TNF-α, IL-8, and IL-17 to IL-10 (Table 4). Lower GA and histologic chorioamnionitis were independent predictors of increased cord blood levels of IL-17 and/or MMP-8, respectively. The impact of preterm premature rupture of membranes >12 h on IL-8/IL-10 ratios in cord blood was of borderline significance (Table 4).

**Table 4:** Inflammatory markers associated with positive *Ureaplasma* screening adjusted for confounding variables.

**Inflammatory marker**	**Predictor**	**Regression coefficient *b* (95% CI)**	***p-*value^a^**
I/T ratio	Presence of *Ureaplasma* spp.	0.394 (0.082 to 0.707)	0.014^*^
	Vaginal delivery	0.063 (−0.069 to 0.194)	0.343
	Vaginal *Ureaplasma* spp.	0.167 (0.051 to 0.283)	0.626
	Vaginal GBS	0.185 (0.004 to 0.279)	0.271
	Vaginal *Candida* spp.	−0.098 (−0.256 to 0.06)	0.218
IL-17^b^	Presence of *Ureaplasma* spp.	0.159 (0.021 to 0.298)	0.025^*^
	GA	−0.035 (−0.07 to −0.001)	0.044^*^
	Vaginal delivery	0.116 (−0.024 to 0.256)	0.104
	Histologic chorioamnionitis	0.096 (−0.032 to 0.224)	0.138
IL-10^b^	Presence of *Ureaplasma* spp.	−0.767 (−1.582 to −0.010)	0.043^*^
	Vaginal delivery	0.192 (−0.502 to 0.980)	0.65
	Vaginal *Ureaplasma* spp.	−0.101 (−0.852 to 0.711)	0.821
	Vaginal *Candida* spp.	−0.214 (−1.15 to 0.722)	0.50
MMP-8^b^	Presence of *Ureaplasma* spp.	0.77 (0.104 to 1.436)	0.020^*^
	GA	−0.272 (−0.439 to −0.104)	0.002^**^
	Vaginal delivery	0.281 (−0.493 to 1.054)	0.471
	Vaginal *Ureaplasma* spp.	0.345 (−0.328 to 1.019)	0.31
	Vaginal *Candida* spp.	−0.494 (−1.296 to 0.308)	0.223
	Antenatal antibiotics	0.463 (−0.237 to 1.162)	0.191
	Histologic chorioamnionitis	1.026 (0.411 to 1.641)	0.001^**^
Ratio TNF-α/IL-10^b^	Presence of *Ureaplasma* spp.	1.249 (0.441 to 2.056)	0.003^**^
	Vaginal delivery	−0.01 (−0.763 to 0.742)	0.978
	Vaginal *Candida* spp.	−0.121 (−0.998 to 0.757)	0.757
Ratio IL-8/IL-10^b^	Presence of *Ureaplasma* spp.	1.309 (0.293 to 2.325)	0.012^*^
	Vaginal delivery	0.116 (−0.024 to 0.256)	0.256
	Vaginal *Ureaplasma* spp.	0.016 (−0.637 to 0.67)	0.961
	Vaginal *Candida* spp.	−0.399 (−1.463 to 0.665)	0.457
	PPROM > 12 h	2.336 (−0.007 to 4.68)	0.051
Ratio IL-17/IL-10^b^	Presence of *Ureaplasma* spp.	1.794 (0.818 to 2.77)	< 0.001^***^
	Vaginal delivery	0.135 (−0.75 to 1.019)	0.762
	Vaginal *Ureaplasma* spp.	0.04 (−0.706 to 0.827)	0.928
	Vaginal *Candida* spp.	−0.399 (−1.463 to 0.665)	0.457

a *Stepwise multi-variate linear regression analyses*.

b *Transformed logarithmically (LN(x))*.

*CI, confidence interval; GA, gestational age*.

* p < 0.05,

** p < 0.01,

*** *p < 0.001*.

## Discussion

With improving care options but enduring occurrence of relevant complications in very immature preterm infants, it is of considerable importance to elucidate whether *Ureaplasma* spp. are harmless commensals or true pathogens in this cohort (Volgmann et al., 2005; Glaser and Speer, 2015). In the present study, 39% of infants <30 weeks' GA and <1,500 g were positive for *Ureaplasma* at initial screening. Detection of *Ureaplasma* spp., however, was not associated with BPD development, rejecting our primary hypothesis. In contrast, we found an increased risk of BPD in *Ureaplasma*-positive infants co-exposed to mechanical ventilation ≥5 days. Positive screening for *Ureaplasma*, moreover, was associated with increased odds of LOS and imbalanced cytokine ratios pushed toward pro-inflammation.

There is a number of epidemiologic data indicating an association between *Ureaplasma* respiratory tract colonization and the development of BPD (Viscardi and Hasday, 2009; Lowe et al., 2014). In contrast, several other studies did not confirm this correlation on adjusted analyses (van Waarde et al., 1997; Patterson et al., 1998; Sung et al., 2011; Payne et al., 2012). In the present investigation, we observed high overall rates of BPD, and we can only speculate on the reasons. With low GA and low birth weight being strong predictors of BPD (Stoll et al., 2010; Isayama et al., 2012; Jobe, 2016), enrolled infants were at increased risk of developing BPD *per se*. The observed BPD rates may further owe to the inclusion of those infants who received supplemental oxygen for ≥28 days but were breathing room air at 36 weeks' postmenstrual age. Depending on the characteristics of a study cohort and the underlying definition of BPD, reported incidences may vary significantly (Lowe et al., 2014). Rates of BPD, defined as oxygen supplementation at 36 weeks' postmenstrual age, ranged from 10 to 25% among preterm infants < 32 weeks' GA born in different regions of Central Europe (Gortner et al., 2011), and ranged from 12 to 15% among VLBW infants followed in the Canadian, Israeli and Japanese neonatal networks (Isayama et al., 2012; Klinger et al., 2013). In contrast, BPD rates were as high as 68% in the NICHD Neonatal Research Network, when including infants who were breathing room air at 36 weeks' postmenstrual age (Stoll et al., 2010). It is noteworthy, however, that we observed a high number of ventilation days in the present study, most likely, due to the restricted use of early non-invasive respiratory support. Mechanical ventilation has been acknowledged as a major postnatal determinant of BPD (Jobe and Bancalari, 2001; Jobe, 2016), and was an independent predictor of BPD in our study. Although both groups did not differ in terms of ventilation days, a particularly strong impact of mechanical ventilation may have masked the effects of less strong predictors.

In contrast to *Ureaplasma* exposure alone, co-exposure to *Ureaplasma* spp. and mechanical ventilation ≥5 days was associated with BPD outcome. These data support previous reports of increased risk of moderate/severe BPD in preterm infants with *Ureaplasma*-positive tracheal aspirates or gastric fluid specimens and mechanical ventilation for any duration and mechanical ventilation ≥14 days, respectively (Sung et al., 2011; Inatomi et al., 2012). Data from a preterm baboon model suggest that antenatal *Ureaplasma* exposure contributes to early lung fibrosis in conjunction with postnatal mechanical ventilation (Viscardi et al., 2006). There is substantial evidence that mechanical ventilation may interact with antenatal and/or postnatal infection to further increase the risk of BPD in preterm infants (Van Marter et al., 2002; Lahra et al., 2009; Balany and Bhandari, 2015). Our findings suggest that early life exposure to *Ureaplasma* spp. and prolonged mechanical ventilation may synergistically contribute to the development of BPD. Of note, according to the present data, co-exposure to *Ureaplasma* spp. and mechanical ventilation ≥5 days may also increase the risk of high-grade IVH in very immature preterm infants. Several case reports and some former studies suggested an implication of *Ureaplasma* spp. in the pathogenesis of IVH and adverse neurologic outcome (Viscardi et al., 2008; Berger et al., 2009; Kasper et al., 2011; Glaser and Speer, 2015), with neonatal systemic and central nervous system inflammation being potential underlying mechanisms (Viscardi et al., 2008; Glaser and Speer, 2015). We confirm and extend those works and discuss that *Ureaplasma*-driven inflammation may modulate neonatal respiratory and neurologic outcome in conjunction with a second postnatal injurious hit.

Inflammation has been acknowledged as a common pathway and principle mechanism in the multifactorial pathogenesis of BPD (Groneck et al., 1994; Jobe and Bancalari, 2001; Balany and Bhandari, 2015). While it remains debated whether or not chorioamnionitis represents a risk factor for BPD (Thomas and Speer, 2011; Hartling et al., 2012), persistent perinatal lung inflammation and postnatal infection have been identified as independent determinants (Groneck et al., 1996; Van Marter et al., 2002; Lahra et al., 2009; Balany and Bhandari, 2015). The present study indicates that *Ureaplasma* spp. effectively induce pro-inflammatory immune responses, but might not stimulate anti-inflammatory responses in VLBW infants and, thus, promote cytokine imbalances pushed toward a pro-inflammatory state. Our findings support data from previous studies documenting increased expression of pro-inflammatory mediators in airway secretions, serum and amniotic fluid specimens of preterm infants colonized with *Ureaplasma* spp. (Groneck et al., 1996; Patterson et al., 1998; Viscardi and Hasday, 2009), and data from *in vitro* models documenting *Ureaplasma*-driven pro-inflammatory responses in macrophages and neonatal monocytes (Manimtim et al., 2001; Viscardi and Hasday, 2009; Glaser et al., 2017, 2018a,b). Of note, bronchoalveolar lavage samples of preterm infants with BPD revealed diminished levels of IL-10, indicating a role for imbalanced inflammatory responses in the pathogenesis of BPD (Jones et al., 1996). *In vitro* studies reported missing IL-10 expression and imbalanced cytokine ratios pushed toward pro-inflammation in *Ureaplasma*-stimulated neonatal monocytes (Manimtim et al., 2001; Glaser et al., 2017). The current data may provide additional evidence of a role for *Ureaplasma* in dysregulated neonatal inflammation.

It is of particular interest, that preterm infants positive for *Ureaplasma* were more likely to develop LOS in the present study. In general, very immature preterm infants are at increased risk of LOS for reasons of immature immunity as well as exposure to a variety of partly interwoven risk factors, such as invasive interventions like central venous lines and mechanical ventilation, parenteral nutrition and delayed initiation of enteral feeding, colonization with LOS-associated microorganisms as well as prior antibiotic treatment or comorbidities of prematurity like PDA and NEC (Boghossian et al., 2013; Dong et al., 2019). The present analysis controlled for the majority of these risk factors. Central venous lines, of note, were less present in infants stratified to the *Ureaplasma*-positive study group. Study groups did not differ in terms of initial empiric antibiotic therapy. However, data on antibiotic treatment in the course of hospital stay and results of weekly routine microbiological screening were not assessed. Despite these limitations, the present results may indicate immunomodulatory features of *Ureaplasma* spp. in the event of secondary bacterial infection, that might drive adverse outcome. This hypothesis adds to data from a preterm sheep model and *in vitro* studies. While monocytes derived from animals with acute (7 day) *Ureaplasma* infection showed augmented cytokine responses to secondary stimulation with *E. coli* lipopolysaccharide (LPS) *in vitro*, monocytes isolated from animals with chronic (70 day) infection displayed decreased responsiveness to secondary LPS stimulation (Kallapur et al., 2011). *In vitro* studies of acute *Ureaplasma* infection in neonatal and adult monocytes documented a differential modulation of cytokine responses in the event of co-infection with *E. coli* LPS (Manimtim et al., 2001; Glaser et al., 2017, 2018a). Moreover, *U. parvum* and *U. urealyticum* were shown to alter Toll-like receptor 2 and 4 expression and to suppress antimicrobial peptide expression in human monocytes *in vitro* (Xiao et al., 2014; Glaser et al., 2017). Given our current findings, we speculate that immunomodulatory capacities of *Ureaplasma* may affect preterm immune homeostasis in specific ways, promoting imbalanced neonatal inflammation on the one hand and increasing susceptibility to secondary bacterial infection on the other hand. Time and duration of *Ureaplasma* exposure might be key to the nature of immune alteration. In fact, positive *Ureaplasma* screening at admission may reflect chronic prenatal challenge to the organism in one infant and rather acute exposure in another. Moreover, a phase of *Ureaplasma*-driven inflammation might be followed by a refractory stage. This sequence is known from a transiently altered immune state referred to as “endotoxin tolerance” (López-Collazo and Del Fresno, 2013). Pathogen-pathogen or polymicrobial interaction as well as host genetics or maturity-related host immune function may additionally contribute to individual outcomes.

Detection rates of *Ureaplasma* in the present study are in accordance with colonization rates of 24–52% reported in preterm infants <32 weeks' GA (van Waarde et al., 1997; Waites et al., 2005; Viscardi and Hasday, 2009; Sung et al., 2011; Payne et al., 2012). Discrepancy to clinical studies reporting lower detection rates in preterm infants may be due to differences in study design and study cohorts, site of sampling and, in particular, the diagnostic and statistical methods applied. It was not until the introduction of molecular techniques that detection of *Ureaplasma* spp. improved and detection frequency increased (Oh et al., 2010; Xiao et al., 2010). Sensitivity and specificity has been shown to be much higher for PCR analysis. Culture technique alone often misses a significant amount of *Ureaplasma-positive* samples (Oh et al., 2010). However, given that the screening for *Ureaplasma* was limited to cord blood and nasopharyngeal swabs obtained at admission, this study still may have missed a certain amount of *Ureaplasma*-positive infants. A very recent investigation reported on increased yield of *Ureaplasma* detection by collecting specimens at different time points (Brand et al., 2018).

Few previous studies on *Ureaplasma* colonization in preterm infants indicated an inverse relationship between colonization and GA, with highest detection rates in infants 25–27 weeks and <26 weeks, respectively (van Waarde et al., 1997; Sung et al., 2011). In our cohort, we found similar detection rates independent of GA. Exclusion of infants >30 weeks' GA might have accounted for this discrepancy. Reports on vertical transmission rates of *Ureaplasma* spp. in pregnancy have documented discrepant percentages, ranging from 15 to 88%, and have also pointed toward an inverse relationship with GA (Sánchez and Regan, 1990; Alfa et al., 1995; Chua et al., 1999; Kafetzis et al., 2004). In the present study, maternal cultures were not subject of speciation and serovar differentiation. Conclusions on potential transmission rates could not be provided.

So far, little is known about differences in virulence at the level of *Ureaplasma* species or among individual serovars (Viscardi and Hasday, 2009; Silwedel et al., 2017; Sweeney et al., 2017). Moreover, there is only scarce data on virulence factors potentially related to disease manifestation (Abele-Horn et al., 1997; Sung et al., 2011; Xiao et al., 2011a; Paralanov et al., 2012; Uchida et al., 2013; Payne et al., 2014; Sweeney et al., 2017). While *U. parvum* has been associated with chorioamniotis and preterm labor in some investigations (Robertson et al., 1986; Kim et al., 2003; Cox et al., 2016; Sweeney et al., 2017), other studies failed to identify serovars more often associated with adverse pregnancy outcome and invasive urogenital tract diseases (Xiao et al., 2011a; Paralanov et al., 2012). A number of studies in preterm infants at risk for BPD documented a predominance of *U. parvum* in clinical isolates (Katz et al., 2005; Oue et al., 2009; Payne et al., 2010, 2012, 2014; Sung et al., 2011; Winters et al., 2013). Predominance of *U. parvum* in the current study is in accordance with these previous observations. There is only one study reporting on a predominance of *U. urealyticum* and an increased risk of BPD in colonized infants (Abele-Horn et al., 1997). In another study, simultaneous colonization with both species was more frequent in preterm infants with the later development of BPD (Katz et al., 2005). So far, no other study has identified an association between neonatal outcome and isolation of a particular *Ureaplasma* species or serovar. The small number of *U. urealyticum*-positive infants in our cohort did not allow to test for differences in neonatal outcome at the species level. However, serovar 3 and 6 accounted for >90% of *U. parvum* swab and serovar 1 for >60% of cord blood isolates. While serovar 6 was not isolated from cord blood, serovar 14 was exclusively found in those specimens. This study is the first to report on potential differences of *U. parvum* serovar distribution in swab and blood samples. In one previous study in preterm infants assessing respiratory tract specimens, serovar 3 and 6 accounted for 96% of *U. parvum* respiratory isolates (Sung et al., 2011). Further data are essential to gain a better understanding of individual serovars and potential associations with colonization and/or invasive infection. As far as maternal colonization with *U. parvum* serovars is concerned, serovar 3 and serovar 6 have been associated with adverse pregnancy outcome, in particular (Robertson et al., 1986; Xiao et al., 2011b). Comparative genome analysis approaches, so far, have been complicated by the limited knowledge of virulence factors in *Ureaplasma per se*, phenomens of size and phase variation in the major virulence factor known, the so-called multiple banded antigen, and a horizontal gene transfer oberserved in *Ureaplasma* (Xiao et al., 2011a; Paralanov et al., 2012; Silwedel et al., 2017; Sweeney et al., 2017).

Strengths of this study include its prospective character and its focus on very immature preterm infants, the assessment of *Ureaplasma* screening by means of culture and PCR technique and the differentiation of species and serovars. To the best of our knowledge, this is the first study to describe the distribution of *U. parvum* serovars in a cohort of VLBW infants in Central Europe and Poland, respectively. Limitations of the present study are given by its monocentric character. Multicentre studies are warranted to confirm our current findings. Further limitations are related to restrictions of secondary endpoint and subgroup analyses. These findings need confirmation by larger studies powered to assess the individual outcome measures.

In conclusion, our data may add considerably to the understanding of *Ureaplasma*-driven neonatal morbidity, suggesting that perinatal *Ureaplasma* exposure may be a driver in the development of neonatal inflammation and infection as well as lung injury. Key might be the duration and intensity of exposure and the addition of a second postnatal injurious hit.

## Data Availability

All datasets generated for this study are included in the manuscript and/or the Supplementary Files.

## Ethics Statement

Prior to enrollment, parents were informed about all details of the study and written parental consent was obtained. The study was approved by the ethics committees of the Poznan University of Medical Sciences (#328/14) and the Medical Faculty of Wuerzburg (#2014081001) and was conducted in accordance with the World Medical Association Declaration of Helsinki.

## Author Contributions

All authors have contributed significantly. KG, AG-L, MS-B, CS: study conception and design; KG, AG-L, NK-L, BH, AW-G: acquisition and analysis of data; KG, AG-L, MS-B, BH, CS: interpretation of data; KG, AG-L, MS-B, NK-L, BH, AW-G, CS: drafting and critical revision; KG, AG-L, MS-B, NK-L, BH, AW-G, CS: final approval; KG, AG-L, MS-B, NK-L, BH, AW-G, CS: agreement to be accountable for all aspects of the work. No assistance was used in the preparation of the manuscript.

### Conflict of Interest Statement

The authors declare that the research was conducted in the absence of any commercial or financial relationships that could be construed as a potential conflict of interest.
